# Circadian variations in clinical symptoms and concentrations of inflammatory cytokines, melatonin, and cortisol in polymyalgia rheumatica before and during prednisolone treatment: a controlled, observational, clinical experimental study

**DOI:** 10.1186/s13075-016-1072-4

**Published:** 2016-07-26

**Authors:** Henrik Galbo, Lisbeth Kall

**Affiliations:** Institute for Inflammation Research, Department of Rheumatology, Rigshospitalet, Copenhagen University Hospital, Blegdamsvej 9, Copenhagen, DK-2100 Denmark

**Keywords:** Inflammation, Autoimmune disease, Cytokines, Muscle disease, Glucocorticoids, Pineal gland, Chronobiology, Chronotherapy

## Abstract

**Background:**

In contrast to rheumatoid arthritis (RA), no systematic investigation of diurnal variation has been carried out in polymyalgia rheumatica (PMR). The aim of the study was to provide the often-requested documentation of the 24-h time course of clinical symptoms in PMR and relate them to concentrations during the day of melatonin, inflammatory cytokines, and cortisol. Furthermore, the effects of 14 days of prednisolone treatment were studied.

**Methods:**

Ten glucocorticoid-naive patients newly diagnosed with PMR and seven non-PMR control subjects were studied for 24 h before treatment and during the 14th day of treatment with 20 mg/day of prednisolone. Global pain and generalized muscle stiffness were monitored by using visual analogue scales, and blood was drawn repeatedly.

**Results:**

In untreated patients, pain and stiffness peaked in the early morning, showing a plateau between 04:00 and 08:00, and then declined to a nadir at 16:00 (2*P* < 0.05). Plasma concentrations of interleukin (IL)-6, IL-8, tumor necrosis factor (TNF)-α, IL-1β, and IL-4 varied with time in both groups (2*P* < 0.05) and peaked between 04:00 and 08:00. Furthermore, except for IL-1β, concentrations of these cytokines and of IL-10 were higher throughout the 24-h observation period in patients than in control subjects (2*P* < 0.05). Also, melatonin and cortisol were consistently higher in patients (2*P* < 0.05) and varied with time (2*P* < 0.05), peaking around 02:00 and 08:00, respectively. In patients, prednisolone abolished symptoms, normalized C-reactive protein, and reduced melatonin, IL-6, IL-8, and TNF-α concentrations (2*P* < 0.05), while IL-10 increased between 10:00 and 14:00.

**Conclusions:**

In PMR, key symptoms show diurnal variation. Furthermore, in PMR, concentrations of melatonin, several pro- and anti-inflammatory cytokines, and cortisol are increased throughout the day and show diurnal variation, as also seen in healthy subjects. The time courses and the inhibitory effects of prednisolone indicate that in PMR, as proposed for RA, melatonin stimulates cytokine production, which in turn accounts at least partly for the symptoms. Furthermore, overall, cortisol may downregulate cytokine production and symptoms. Stimulation of IL-10 secretion may participate in the anti-inflammatory effects of prednisolone. These findings support use of chronotherapy in PMR and encourage study of circadian variations in other inflammatory autoimmune diseases.

**Electronic supplementary material:**

The online version of this article (doi:10.1186/s13075-016-1072-4) contains supplementary material, which is available to authorized users.

## Background

In rheumatoid arthritis (RA), key clinical symptoms and findings show a circadian variation, with more prominent joint swelling, stiffness, and pain occurring in the early morning [1, 2]. Interestingly, within recent years, it has been pointed out that, in RA, the diurnal variation in symptoms coincides with and may be partly mediated by a circadian variation in plasma concentrations of proinflammatory cytokines [1–5]. Furthermore, it has been proposed that, in RA, the pineal hormone and key chronobiological marker melatonin regulates circadian cytokine production, and it has been shown that plasma concentrations of melatonin are higher in patients with RA than in control subjects and peak a couple of hours before cytokine concentrations [1–3]. However, cortisol, which has a response to light that is opposite that of melatonin and, accordingly, a different diurnal time course, may counteract the inflammatory processes [1, 4].

Polymyalgia rheumatica (PMR) is the most common chronic autoimmune rheumatic disease of the elderly [6, 7]. For the most part, it is easily distinguished from RA. However, in PMR, morning stiffness and aching are also generally accepted core symptoms, as reflected by the various sets of criteria defined for its diagnosis [4, 6–10]. Nevertheless, although a circadian rhythm seems obvious, no systematic investigation of the 24-h variation of pain and stiffness in PMR has been published, and such data are often requested [5, 11]. Furthermore, while in both PMR [9, 12, 13] and RA [14] plasma concentrations of many cytokines, when measured at a single time point in the morning, have been found to be higher than in healthy subjects, in PMR the circadian courses of cytokines and cortisol have not yet been determined in controlled studies [11, 15]. Also, in contrast to RA, melatonin concentrations have not been evaluated in PMR.

Thus, to add to the existing clinical evidence and to elucidate possible pathophysiological mechanisms in PMR, in the present study clinical symptoms and plasma concentrations of cytokines, melatonin, and cortisol were monitored for 24 h in patients with PMR and healthy subjects. Both groups were studied before and after 13 days of glucocorticoid (GC) treatment, which is known to abolish symptoms in patients with PMR [6–9, 12, 13, 16].

## Methods

### Subjects

Ten consecutively referred GC-naive patients newly diagnosed with PMR according to the criteria proposed by Chuang and colleagues [6, 7, 10, 17], as well as seven non-PMR control subjects matched according to sex, age, and body mass index (BMI), were recruited by advertising and included in the study after a standard medical examination and a comprehensive blood and urine screening (Table 1). Subjects were studied between October 2011 and October 2012. It follows that we were unable to use the most recent PMR criteria, which were published in April 2012 [10]. However, the latter criteria are still provisional and awaiting further validation, and, in the most recent reviews of PMR, the Chuang criteria are mentioned on a par with the newer provisional criteria [6, 7, 10, 16]. In the nature of the case, the two criteria sets are very similar, but the fact that the demand for a high sedimentation rate is stricter in the Chuang criteria implies that the patients in the present study would also be accepted with the new criteria. The sample size was calculated by estimating standardized effect sizes from reported diurnal changes in pain and stiffness in RA and on the basis of our own experience regarding the SD of similar visual analogue scale (VAS) measurements in RA as well as our previous cytokine measurements with the applied assays in PMR (e.g., [9, 12, 13]). With an α level of 0.05 and a statistical power of 80 %, a minimum sample size of seven to ten patients with PMR was determined.

**Table 1:** Subject characteristics

	Patients with PMR (*n* = 10)	Control subjects (*n* = 7)
Sex, female/male	6/4	4/3
Age, years	76 ± 2.2	71 ± 1.6
BMI, kg∙m^−2^	24.0 ± 1.3	26.7 ± 1.2
Blood pressure (systolic/diastolic, mmHg)		
Before treatment	143 ± 7/88 ± 6	141 ± 10/86 ± 6
After treatment	147 ± 10/81 ± 3	144 ± 9/85 ± 6
ESR, mm/h		
Before treatment	72 ± 8^a^	10 ± 2
After treatment	21 ± 4^b^	8 ± 1
CRP, mg/L		
Before treatment	56 ± 7^a^	3 ± 1
After treatment	7 ± 2^b^	1 ± 0.3
PMR-AS		
Before treatment	38 ± 2^a^	0.2 ± 0.1
After treatment	5 ± 1^b^	0 ± 0

*Abbreviations: BMI* body mass index, *CRP* C-reactive protein, *ESR* erythrocyte sedimentation rate, *PMR* polymyalgia rheumatica, *PMR-AS* polymyalgia rheumatica activity score [18]

Values are mean ± SEM. Subjects were studied before treatment and during the 14th day of prednisolone treatment (20 mg/day)

^a^ Patients vs control subjects, 2*P* < 0.05

^b^ After vs before treatment, 2*P* < 0.05

None of the subjects fulfilled the exclusion criteria: prior or current use of GCs or other immunosuppressive drugs; signs of giant cell arteritis, including persistent headache, visual disturbances, jaw claudication, abnormal pulsation or wall of temporal artery, and scalp tenderness; inflammatory diseases other than PMR; infections with systemic impact; positive blood or urine culture; uncontrolled diabetes mellitus or hypertension; neuromuscular disease; disturbance of calcium homeostasis; or prior cancer. In both groups, some subjects, as expected for their age, were taking medications for, for example, hypertension, hypercholesterolemia, type 2 diabetes, acid regurgitation, bladder hyperactivity, or prostatic hyperplasia. Because these medications had no known impact on study variables, they were continued. Only paracetamol was allowed as an analgesic, but intake was stopped the evening before hospitalization. None of the subjects smoked during the study.

### Study design

The study was carried out in compliance with the Declaration of Helsinki and was approved by the regional ethics committee of Copenhagen (approval number H-1-2011-060). Informed written consent was obtained before a subject was included. Both patients and control subjects were admitted to Rigshospitalet, Copenhagen, for 24 h before treatment and on the 14th day of GC treatment with prednisolone (20 mg taken at 08:00 [6, 7, 16]). Usually, study of a patient with PMR was followed by study of a matched control subject. No subjects were studied in December and January or in June and July. These facts would minimize any influence of season on the study outcome. However, of even greater importance in this respect, light conditions in this hospital are controlled and are the same all year round. On both admissions, subjects arrived at the hospital at 10:00. Between 10:00 and 10:30, a small Teflon catheter was inserted into a forearm vein for blood sampling at 11:00, 12:00, 14:00, 16:00, 19:00, 21:00, 22:00, 23:00, 00:00; 01:00, 02:00, 03:00, 04:00, 05:00, 06:00, 06:30, 07:15, 08:00, 09:00, and 10:00. When necessary at nighttime, a small pencil lamp, directed away from the patient’s face, was used during the brief blood sampling. However, mostly it was possible to take the samples while the patients were sleeping. Otherwise, they were told to close their eyes during the sampling. Symptoms of global pain and generalized muscle stiffness were assessed by the patients and the physician using a VAS (0–10 cm) at 12:00, 16:00, 20:00, 04:00, and 08:00. To limit the impact on the sleeping pattern of the subjects, VAS scores were not collected from 20:00 to 04:00. The Polymyalgia Rheumatica Activity Score (PMR-AS) was assessed once in the morning before and after treatment [12, 18]. Meals were served at 8:00, 12:00, and 18:00. Subjects were ambulant in the daytime.

### Blood samples

Blood was drawn in ethylenediaminetetraacetic acid tubes and rapidly centrifuged at 1200 rpm and room temperature for 2 minutes. Plasma was stored at −80 °C until analysis within 6 months. In separate stock Vacutainer tubes (BD Biosciences, San Jose, CA, USA), blood was drawn for erythrocyte sedimentation rate (ESR) measurement.

### Analytical methods

Interleukin (IL)-6, IL-8, tumor necrosis factor (TNF)-α, IL-10, IL-1β, and IL-4 were measured using a multiplex immunoassay method on a Luminex 100 platform (Luminex, Austin, TX, USA) with specific, high-sensitivity assay kits (MILLIPLEX high-sensitivity human cytokine magnetic bead kit, HSCYTMAG-60SK HSCYTMAG60SPMX13 [premixed]; EMD Millipore, Billerica, MA, USA). Standard curves were prepared in plasma-analogous matrix contained in the assay kit. Generally, samples from a patient and a control subject were analyzed simultaneously, and all assays were carried out in duplicate and by the same technician. The ranges of intra- and interassay coefficients of variation (coefficient of variation [CV], calculated from single determinations) for the cytokine analyses were 7.7–10.6 % and 9.4–15.2 %, respectively. Melatonin was measured by radioimmunoassay with intra- and interassay CVs of 10 % and 11 %, respectively (BA R-3300; LDN GmbH, Nordhorn, Germany). Cortisol, with intra- and interassay CVs of 6.3 % and 10.4 %, respectively, and cross-reactivity of prednisolone of 4.4 % (KGE008; R&D Systems, Minneapolis, MN, USA), and C-reactive protein (CRP), with intra- and interassay CVs of 8.3 % and 6.6 %, respectively (Human Quantikine DCRP00; R&D Systems), were determined by enzyme-linked immunosorbent assay. ESR was measured using the Westergren method (BD Sedi-15; BD Biosciences).

### Statistics

Data are expressed as mean ± SEM. In the figures, smoothed curves drawn using Prism analysis based on second-order polynomial and four neighboring points (GraphPad Software, La Jolla, CA, USA) are shown [19]. Statistical analysis was performed using SigmaStat software (Systat Software, San Jose, CA, USA). Values of 2*P* ˂ 0.05 were considered significant in two-tailed testing. Anthropometric data were evaluated by performing *t* tests, unpaired or paired as applicable. Variation with time during the day was evaluated by one-way analysis of variance (ANOVA) with repeated measures, as well as by two-way repeated-measures ANOVA to also evaluate the effect of either disease (PMR) or treatment (prednisolone) [20–22]. Differences were located by performing the Newman-Keuls post hoc test. If compatibility of data with normal distribution could not be confirmed with large probability according to the Shapiro-Wilk *W* test, data were also analyzed by nonparametric testing (using the Friedman ANOVA test and the Wilcoxon and Mann-Whitney *U* ranking tests for paired and unpaired data, respectively). However, these tests yielded exactly the same conclusions as parametric testing, supporting the robustness of the latter.

The total AUC for each of the 24-h observation periods were calculated using the trapezoidal rule, and effects of disease and treatment were evaluated by two-way ANOVA. Furthermore, to reduce effects of random variation of single samples on time course evaluations, AUCs were also calculated for 3- to 6-h subintervals of the day, and effects of time, disease, and treatment were assessed for these subintervals using ANOVA. However, this approach yielded the same conclusions as we obtained from analysis of the raw data. Finally, Pearson’s or nonparametric Spearman’s correlation coefficients were calculated as applicable. To limit the difficult statistical problem of potential mass significance, correlation analyses were not carried out indiscriminately, but only according to decisions made in advance of the study about which relationships would be pertinent to characterize. An additional way to deal with the problem would be to adjust *P* values (e.g., by the Bonferroni method). However, this is known to cause overcorrection of unknown magnitude. So, we report the directly determined *P* values for individual correlations, abstracting from the fact that several analyses were carried out.

## Results

Baseline anthropometrics, comprising age, BMI, body temperature (37.1 ± 0.1 vs 36.7 ± 0.2 °C), heart rate (78 ± 5 vs 66 ± 4 beats/minute), and blood pressure did not differ between untreated patients and control subjects (Table 1). Before treatment, CRP (56 ± 7 vs 3 ± 1 mg/L), ESR, and PMR-AS were significantly higher (2*P* ˂ 0.05) in patients with PMR than in control subjects (Table 1). However, after 13 days of prednisolone treatment, patients had achieved profound clinical remission, as judged by standard physician assessment as well as on the basis of markedly reduced PMR-AS scores (Table 1). Furthermore, after treatment, CRP (7 ± 2 mg/L) and ESR levels had decreased markedly in patients, now being within normal limits in all (CRP) and in six of ten (ESR) subjects, respectively (Table 1).

### Diurnal variation of clinical symptoms

**Fig. 1:**
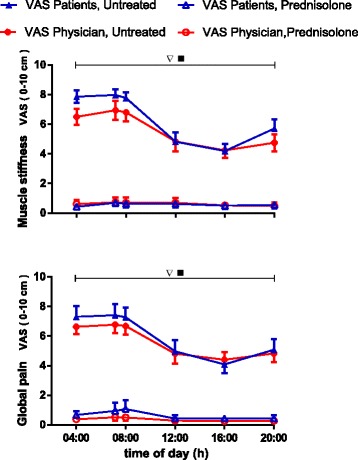
Generalized muscle stiffness and global pain during the day in ten patients with polymyalgia rheumatica. Symptoms were assessed both by the patients and by a physician before treatment and during the 14th day of treatment with 20 mg of prednisolone taken in the morning. Values are mean ± SEM. *Inverted open triangles*, Variation with time in untreated patients (2*P* < 0.05). *Filled squares*, Difference between prednisolone-treated and untreated patients (2*P* < 0.05). *VAS* visual analogue scale

In the patients, both global pain and experience of muscle stiffness attained the highest values in the early morning, showing a plateau between 04:00 and 08:00 (Fig. 1). Subsequently, values decreased significantly (2*P* ˂ 0.05), reaching a nadir at 16:00 (Fig. 1). Assessment by the physician mirrored that of the patients (Fig. 1). After treatment with prednisolone, symptoms were negligible and showed no diurnal variation (Fig. 1).

### Diurnal variation of plasma concentrations of cytokines and cortisol in untreated patients and control subjects

**Fig. 2:**
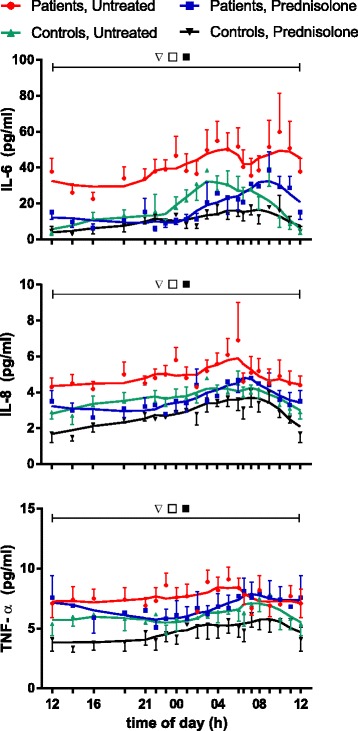
Plasma concentrations of various cytokines monitored for 24 h. Ten patients with polymyalgia rheumatica (PMR) and seven non-PMR control subjects were studied before treatment and during the 14th day of treatment with 20 mg of prednisolone. Values are mean ± SEM. Smoothed curves are included. *Inverted open triangles*, Variation with time in each of the groups (2*P* < 0.05). *Open squares*, Difference between untreated patients and control subjects (2*P* < 0.05). Filled squares, Effect of prednisolone (2*P* < 0.05) in both patients and control subjects. *IL* interleukin, *TNF* tumor necrosis factor

**Fig. 3:**
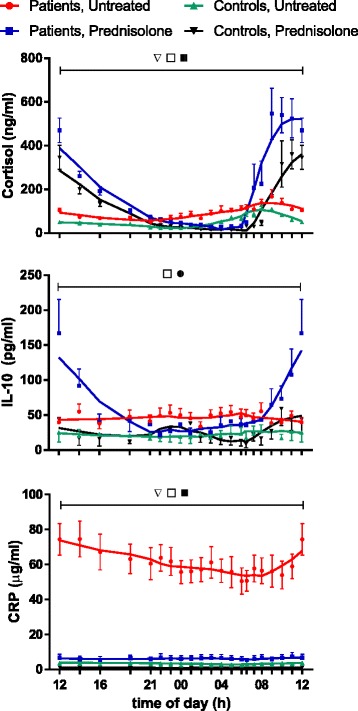
Plasma concentrations of cortisol, interleukin (IL)-10, and C-reactive protein (CRP) monitored for 24 h. Ten patients with polymyalgia rheumatica (PMR) and seven non-PMR control subjects were studied before treatment and during the 14th day of treatment with 20 mg of prednisolone. Values are mean ± SEM. Smoothed curves are included. *Inverted open triangles*, Variation with time in each of the groups (2*P* < 0.05), except for CRP, in prednisolone-treated patients (not significant). *Open squares*, Difference between untreated patients and control subjects (2*P* < 0.05). *Filled squares*, Effect of prednisolone (2*P* < 0.05) in both patients and control subjects. *Filled circle*, Effect of time only in prednisolone-treated groups (interaction) (2*P* < 0.05)

**Fig. 4:**
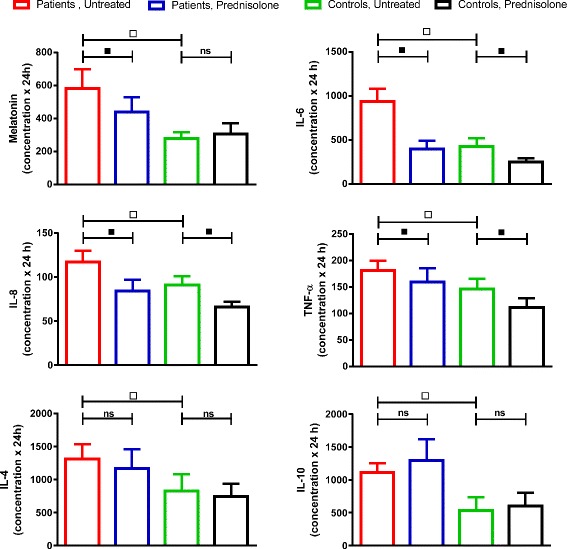
Total plasma concentration AUCs for the 24-h observation periods (24-h AUCs). Ten patients with polymyalgia rheumatica (PMR) and seven non-PMR control subjects had melatonin and various cytokines measured before treatment and during the 14th day of treatment with 20 mg of prednisolone. Values are mean ± SEM. *Open squares*, Difference between untreated patients and control subjects (2*P* < 0.05). *Filled squares*, Effect of prednisolone (2*P* < 0.05). *IL* interleukin

Concentrations of IL-6, IL-8, and TNF-α (Fig. 2), as well as of IL-4 and IL-1β (see Additional file 1), but not of IL-10 (Fig. 3), varied significantly with time in both groups (2*P* ˂ 0.05). They peaked in the early morning between 04:00 and 08:00. Furthermore, concentrations of all of the above-mentioned cytokines except IL-1β were significantly higher (2*P* ˂ 0.05) throughout the 24-h observation period in patients than in control subjects. Consequently, 24-h AUCs for all of the cytokines except IL-1β were significantly larger in patients than in control subjects (2*P* ˂ 0.05) (Fig. 4 and Additional file 2).

Cortisol and CRP concentrations also showed diurnal variation in both groups and were significantly higher in patients than in control subjects (2*P* ˂ 0.05) (Fig. 3). Correspondingly, 24-h AUCs for cortisol were 2137 ± 184 ng/ml × 24 h (patients) and 1253 ± 144 ng/ml × 24 h (control subjects) (2*P* ˂ 0.004). However, while cortisol peaked around 08:00, CRP reached a nadir at this time point (Fig. 3).

### Effects of prednisolone on cytokines and cortisol in plasma

During the 14th day of prednisolone treatment, IL-6, IL-8, and TNF-α concentrations in both patients and control subjects were lower than before treatment (2*P* ˂ 0.05) (Fig. 2). Consequently, 24-h AUCs were also diminished (2*P* ˂ 0.05) (Fig. 4). Concentrations of CRP were also reduced by prednisolone in the patients (2*P* ˂ 0.05) (Fig. 3), whereas levels of IL-4 and IL-1β were not significantly affected (see Additional files 1 and 2). For cortisol and IL-10 concentrations, an interaction between prednisolone and time was found in both groups, resulting in significantly increased levels from about 10:00 to 14:00 (2*P* ˂ 0.05) (Fig. 3).

### Melatonin

**Fig. 5:**
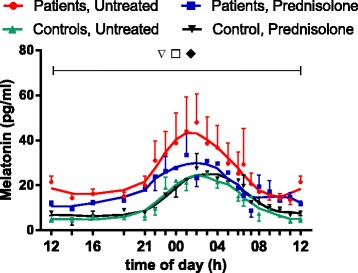
Plasma concentrations of melatonin monitored for 24 h. Ten patients with polymyalgia rheumatica (PMR) and seven non-PMR control subjects were studied before treatment and during the 14th day of treatment with 20 mg of prednisolone. Values are mean ± SEM. Smoothed curves are included. *Inverted open triangle*, Variation with time in each of the groups (2*P* < 0.05). *Open square*, Difference between untreated patients and control subjects (2*P* < 0.05). *Filled diamond*, Effect of prednisolone in the patients (2*P* < 0.05)

In both patients and control subjects, regardless of whether they were treated with prednisolone, melatonin concentrations showed significant diurnal variation (2*P* ˂ 0.05) and peaked around 02:00, about 4 h earlier than cytokine concentrations (Fig. 5). In untreated patients, concentrations were significantly higher than in control subjects (2*P* ˂ 0.05) and were also reduced by prednisolone treatment (2*P* ˂ 0.05) (Figs. 4 and 5).

### Correlations

The TNF-α peak and 24-h AUC concentrations both correlated with the peak in patient-reported pain (*r* = 0.6, 2*P* ˂ 0.08, and *r* = 0.7, 2*P* ˂ 0.03, respectively) as well as with the peak in patient-reported stiffness (*r* = 0.6, 2*P* ˂ 0.08, *r* = 0.8, 2*P* ˂ 0.006, respectively). The IL-6 24-h AUC correlated with the average patient-reported pain level during 04:00–08:00 (*r* = 0.6, 2*P* ˂ 0.05). The IL-8 24-h AUC correlated with the physician-estimated peak pain (*r* = 0.6, 2*P* ˂ 0.05). IL-10 did not correlate significantly with symptoms. The melatonin peak and 24-h AUC concentrations correlated with the TNF-α average level during 04:00–06:00 in patients (*r* = 0.7, 2*P* ˂ 0.04, and *r* = 0.6, 2*P* ˂ 0.07, respectively) and in patients and control subjects combined (*r* = 0.6, 2*P* ˂ 0.01, and *r* = 0.5, 2*P* ˂ 0.03, respectively). Various measures of cortisol secretion correlated significantly with measures of IL-6 secretion, but not of TNF-α secretion, in patients as well as in patients and control subjects combined (e.g., peak concentration vs peak concentration and 24-h AUC vs 24-h AUC, *r* = 0.8 and 2*P* ˂ 0.006 for all).

## Discussion

In the present study, several new important findings that extend knowledge about PMR were obtained. First, the previously presumed diurnal variation in key symptoms has now been documented. In untreated patients pain and stiffness peak between 04:00 and 08:00 and then decline to a nadir at 16:00. Second, the diurnal variation in symptoms is paralleled by circadian changes in plasma concentrations of several proinflammatory and anti-inflammatory cytokines, most of which are increased compared with findings in healthy subjects. Third, plasma melatonin and cortisol concentrations are increased in patients with PMR throughout the day. Furthermore, possibly reflecting causal relationships, while the peak in melatonin precedes the peak in cytokine concentrations and symptoms by about 4 h, these variables decline when cortisol peaks. Finally, after 13 days of prednisolone treatment, abolition of symptoms in patients with PMR is accompanied by marked reductions in melatonin and cytokine levels around the clock.

It has previously been shown that the morning plasma concentrations of the proinflammatory cytokines TNF-α, IL-6, and IL-8 are higher, whereas concentrations of IL-1β are similar, in patients with PMR compared with non-PMR control subjects [9, 12–14]. It is a new finding that also concentrations of the anti-inflammatory cytokines IL-4 and IL-10 are increased in patients with PMR. Furthermore, it is a new observation that the above-mentioned differences between patients and control subjects exist throughout the day (Figs 2, 3, 4 and Additional file 1). Previously, lack of agreement between studies regarding the existence of differences in cytokine concentrations between patients with PMR and control subjects was tentatively ascribed to differences in sampling time [11]. However, according to the present findings, this explanation cannot be sustained. Physical activity is known to increase concentrations of several cytokines in plasma [23, 24]. However, differences in physical activity would not explain the differences in cytokine concentrations between groups in the present study. This is so because during hospitalization the possibilities for exercise were limited, and, if anything, patients with PMR would be supposed to be less active than control subjects.

Not only are plasma concentrations of many cytokines markedly higher in patients with PMR than in control subjects throughout the day, but the present study has also shown that in both groups concentrations show diurnal variation (Fig. 2 and Additional file 1). Previously, diurnal variation was indicated in a study of nocturnal cytokine levels in healthy men, the study reporting that IL-1β and IL-6 were more likely to be above detection limits during sleep than during preceding wakefulness [25]. Furthermore, in a study of patients with PMR with no control subjects, IL-6 showed a diurnal variation comparable to the present findings [26]. In contrast, various other cytokines showed no clear indication of circadian variation [26]. In RA, diurnal variation in cytokine concentrations, albeit less comprehensively than in the present study of PMR, has been reported and proposed to be partly responsible for the circadian rhythm of the clinical symptoms [1, 5, 20]. In the present study, often-requested data [5, 11] documenting a similar diurnal variation in major symptoms of PMR (i.e., pain and stiffness) are presented (Fig. 1). It is an intriguing possibility that also in PMR the circadian variation in these symptoms is to a great extent caused by a parallel variation in cytokine levels. Interestingly, in fibromyalgia, which may be confused with RA and PMR but which is a noninflammatory condition, pain and stiffness do not display circadian rhythms [27].

Melatonin concentrations in plasma have not previously been determined in PMR. We found that they display the same diurnal variation as seen in control subjects (Fig. 5). However, throughout the day, concentrations in patients were around two times higher than in control subjects. Furthermore, they peaked about 4 h before the elevated cytokine concentrations. These findings are in agreement with the proposal based on studies in RA that melatonin is involved in the regulation of the circadian variation in cytokine concentrations [1, 5]. Melatonin has a variety of effects on the immune system, which may differ according to study conditions, such as between cell types and in vivo vs in vitro [28, 29]. However, in accordance with the above interpretation, melatonin administration has recently been shown to enhance the release of both proinflammatory and anti-inflammatory cytokines under inflammatory conditions in rats [30]. Also in agreement with an association between enhanced melatonin secretion and inflammation, in the noninflammatory but painful condition of fibromyalgia, melatonin concentrations are identical to those of healthy subjects [27]. In RA, higher circadian melatonin concentrations in a northern compared with a southern European population were associated with increasing incidence of the disease with latitude [31]. It is interesting to note that also the prevalence of PMR is particularly high in Northern Europe [6, 7]. An obvious hypothesis is that the increased prevalence of autoimmune diseases in Northern Europe is related to the reduced daily light exposure, present at least during wintertime, and consequent increased immunostimulation by melatonin [32].

During the day, the secretion of cortisol is displaced relative to that of melatonin, and the two hormones have many mutually opposite effects on the immune system [1, 33]. Accordingly, in RA, also cortisol has been proposed to participate in the regulation of the diurnal variation in cytokine secretion and symptoms [1, 3, 34]. While in RA cytokine concentrations peaking in the night and early morning may increase cortisol secretion by stimulation of both the hypothalamus and the adrenal medulla, cortisol may in turn downregulate cytokine expression [3, 35, 36]. The present study shows that similar relationships may be at play in PMR. Thus, cytokine concentrations peaked somewhat earlier than cortisol concentrations and declined in parallel with clinical symptoms, when cortisol levels peaked about 08:00 (Figs. 1, 2 and 3). Furthermore, administration of prednisolone, a cortisol analogue, markedly reduced cytokine concentrations and symptoms throughout the day (Figs. 1, 2, 3 and 4). This is in accord with and adds to previous findings obtained in the morning [9, 13, 14]. Our findings also confirm the previous observation that, in PMR, prednisolone treatment diminishes IL-6 peak concentration and 24-h AUC [26]. In the patients with PMR in the present study, prednisolone diminished melatonin concentrations as well (Fig. 5). This is in line with the finding that cortisol inhibits melatonin synthesis in the pineal gland [37].

It has been claimed that, in both RA and PMR, cortisol secretion is inappropriately low in relation to proinflammatory status—“the disproportion principle” [38]—and that this may contribute to the symptoms [3, 11, 36]. Indicating that hypothalamic-pituitary-adrenal axis activation, albeit less than might be expected from the state of inflammation, may nevertheless be present, we previously found higher morning concentrations of both adrenocorticotropic hormone and cortisol in patients with PMR than healthy subjects [39]. The fact that researchers in other studies had not found such differences was explained by lower disease activity and longer disease onset to study duration in those studies [39]. The present study extends our previous findings by showing that, in PMR, cortisol concentrations may be increased throughout the day (Fig. 3), resulting in a doubling of 24-h cortisol AUC in patients compared with control subjects.

In RA, administration of GCs prior to the circadian flare in inflammatory activity has been proven to be advantageous compared with traditional administration in the morning [5, 15, 40–42]. The present finding in PMR of a similar diurnal variation in cytokines and symptoms indicates that also in PMR adaptation of the timing of GC administration to the chronobiology of the disease would optimize the treatment. In fact, preliminary experiments have been reported to show positive outcomes of such an approach, with reductions in IL-6 levels and morning stiffness being more pronounced upon administration of prednisolone in the night than in the morning [26].

Although the cross-reactivity of prednisolone in the cortisol assay is only 4.4 %, the high cortisol levels seen in both patients and control subjects upon prednisolone administration in the morning (Fig. 3) most likely reflect comeasurement of high prednisolone concentrations. Still, previously, increases in early morning peak cortisol concentrations have been found in patients with RA upon nighttime prednisone administration, even after correcting for cross-reactivity by direct measurement of drug levels [22]. It was speculated that the enhanced nocturnal rise in plasma cortisol reflected a change in relationship between the hypothalamic-pituitary-adrenal axis and immune system activation [22].

In the present study, concentrations of the anti-inflammatory cytokine IL-10 also were increased by prednisolone (Fig. 3). On the basis of previous studies of lipopolysaccharide-stimulated IL-10 secretion in whole blood in vitro, it has been concluded that cortisone administration in vivo has little effect on IL-10 secretion [35]. However, the presented data allow the conclusion that GC in fact does enhance IL-10 secretion, because secretion decreased in control experiments but not upon cortisone administration [35]. Albeit not confirmed by our data, it is interesting to note that, on the basis of correlation analysis, a favorable role of IL-10 in PMR has been proposed [43]. CRP can be considered an inflammatory cytokine regulated by circulating IL-6 and other cytokines [44]. In contrast to the view that acute-phase CRP values show no diurnal variation [45], in the present study in untreated patients with PMR, CRP showed a clear circadian rhythm with a nadir at 08:00 (Fig. 3). This time course seems compatible with the delay inherent in intranuclear expression stimulation and in the long plasma half-life of CRP.

## Conclusions

The present study documents diurnal variation of key symptoms in PMR and points out potentially underlying pathophysiological mechanisms. Thus, in PMR concentrations of melatonin, several pro- and anti-inflammatory cytokines and of cortisol are increased throughout the day and show circadian variation, as also seen in healthy subjects. The time courses are compatible with the view that, in PMR, as proposed also for RA, melatonin stimulates cytokine production, which in turn accounts at least partly for the clinical symptoms, while overall cortisol downregulates cytokine production and symptoms. The view is supported by the finding that, in PMR, prednisolone diminishes levels of both melatonin and proinflammatory cytokines while abolishing symptoms throughout the day. In addition, stimulation of IL-10 secretion may to some extent mediate the anti-inflammatory effects of prednisolone. Finally, in untreated PMR, CRP also shows diurnal variation. These findings support future use of chronotherapy in PMR and encourage study of circadian variations in other inflammatory autoimmune diseases.

## Abbreviations

ANOVA, analysis of variance; BMI, body mass index; CRP, C-reactive protein; CV, coefficient of variation; ESR, erythrocyte sedimentation rate; GC, glucocorticoid; IL, interleukin; PMR, polymyalgia rheumatica; PMR-AS, Polymyalgia Rheumatica Activity Score; RA, rheumatoid arthritis; TNF, tumor necrosis factor; VAS, visual analogue scale

## Additional files

Additional file 1: Figure S6. Plasma concentrations of IL-4 and IL-1β monitored for 24 h. Ten patients with PMR and seven non-PMR control subjects were studied before treatment and during the 14th day of treatment with 20 mg of prednisolone. Values are mean ± SEM. Smoothed curves are included. *Open inverted triangles*, Variation with time in all groups (2*P* < 0.05), except for prednisolone-treated control subjects. *Open boxes*, Difference between untreated patients and control subjects (2*P* < 0.05). *Filled diamonds*, Effect of prednisolone in control subjects (2*P* < 0.05). (DOCX 245 kb) Additional file 2: Figure S7. Total areas under the plasma concentration curve for 24-h measurements of IL-1β (24-h AUCs). Ten patients with PMR and seven non-PMR control subjects had IL-1β measured before treatment as well as during the 14th day of treatment with 20 mg of prednisolone. Values are mean + SEM. Filled squares, Effect of prednisolone (2*P* < 0.05). (DOCX 49 kb)
