# Supplementary material for: Circadian variations in clinical symptoms and concentrations of inflammatory cytokines, melatonin, and cortisol in polymyalgia rheumatica before and during prednisolone treatment: a controlled, observational, clinical experimental study
Source: Arthritis Res Ther. 2016 Jul 26;18:174. doi: 10.1186/s13075-016-1072-4 (PMC4960703; doi:10.1186/s13075-016-1072-4)
Supplement: Supplementary file 1 [file 13075_2016_1072_MOESM1_ESM.docx]

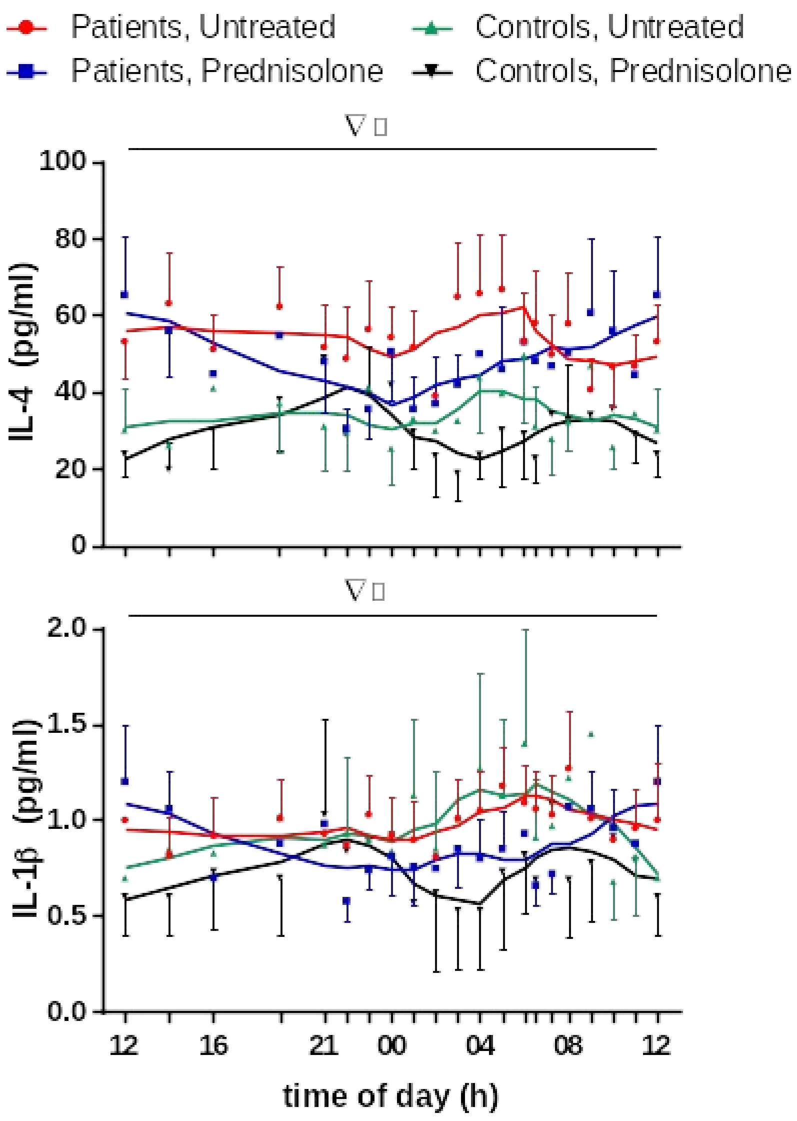

Patients, Untreated
Controls, Untreated
Patients, Prednisolone
Controls, Prednisolone
IL-4 (pg/ml)
IL-1β (pg/ml)
time of day (h)
