# Supplementary material for: Circadian variations in clinical symptoms and concentrations of inflammatory cytokines, melatonin, and cortisol in polymyalgia rheumatica before and during prednisolone treatment: a controlled, observational, clinical experimental study
Source: Arthritis Res Ther. 2016 Jul 26;18:174. doi: 10.1186/s13075-016-1072-4 (PMC4960703; doi:10.1186/s13075-016-1072-4)
Supplement: Supplementary file 2 [file 13075_2016_1072_MOESM2_ESM.docx]

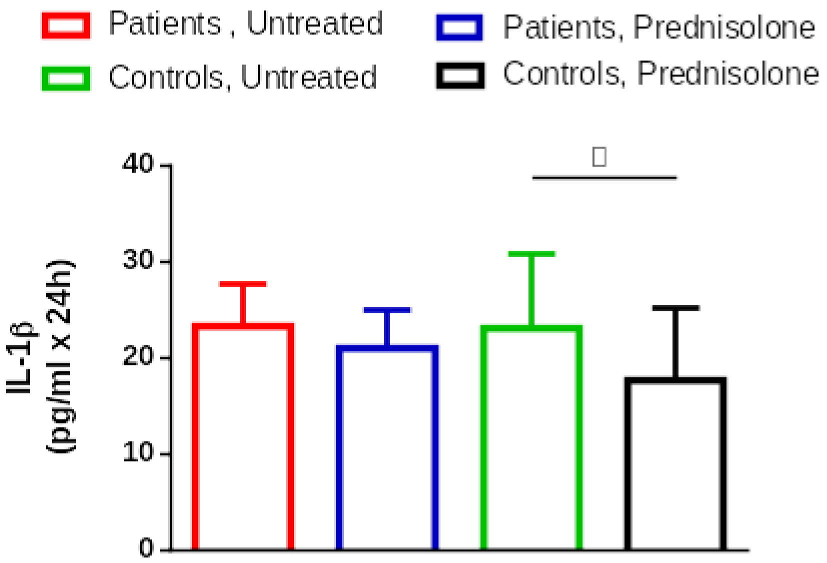
Patients , Untreated
Patients, Prednisolone
Controls, Untreated
Controls, Prednisolone
IL-1β
(pg/ml x 24h)
40
30
20
10
0
